# Supplementary material for: Replaying germinal center evolution on a quantified affinity landscape
Source: Cell. Author manuscript; Available in PMC 2026 Jul 14. (PMC13360575; doi:10.1016/j.cell.2026.05.013)
Supplement: MMC1 [file NIHMS2177267-supplement-MMC1.pdf]

Cell, Volume *189*

Supplemental information

Replaying germinal center evolution

on a quantified affinity landscape

William S. DeWitt, Ashni A. Vora, Tatsuya Araki, Jared G. Galloway, Tanwee Alkutkar, Juliana Bortolatto, Tiago B.R. Castro, Will Dumm, Chris Jennings-Shaffer, Tongqiu Jia, Luka Mesin, Gabriel Ozorowski, Juhee Pae, Duncan K. Ralph, Jesse D. Bloom, Armita Nourmohammad, Yun S. Song, Andrew B. Ward, Tyler N. Starr, Frederick A. Matsen IV, and Gabriel D. Victora

Supplemental Information

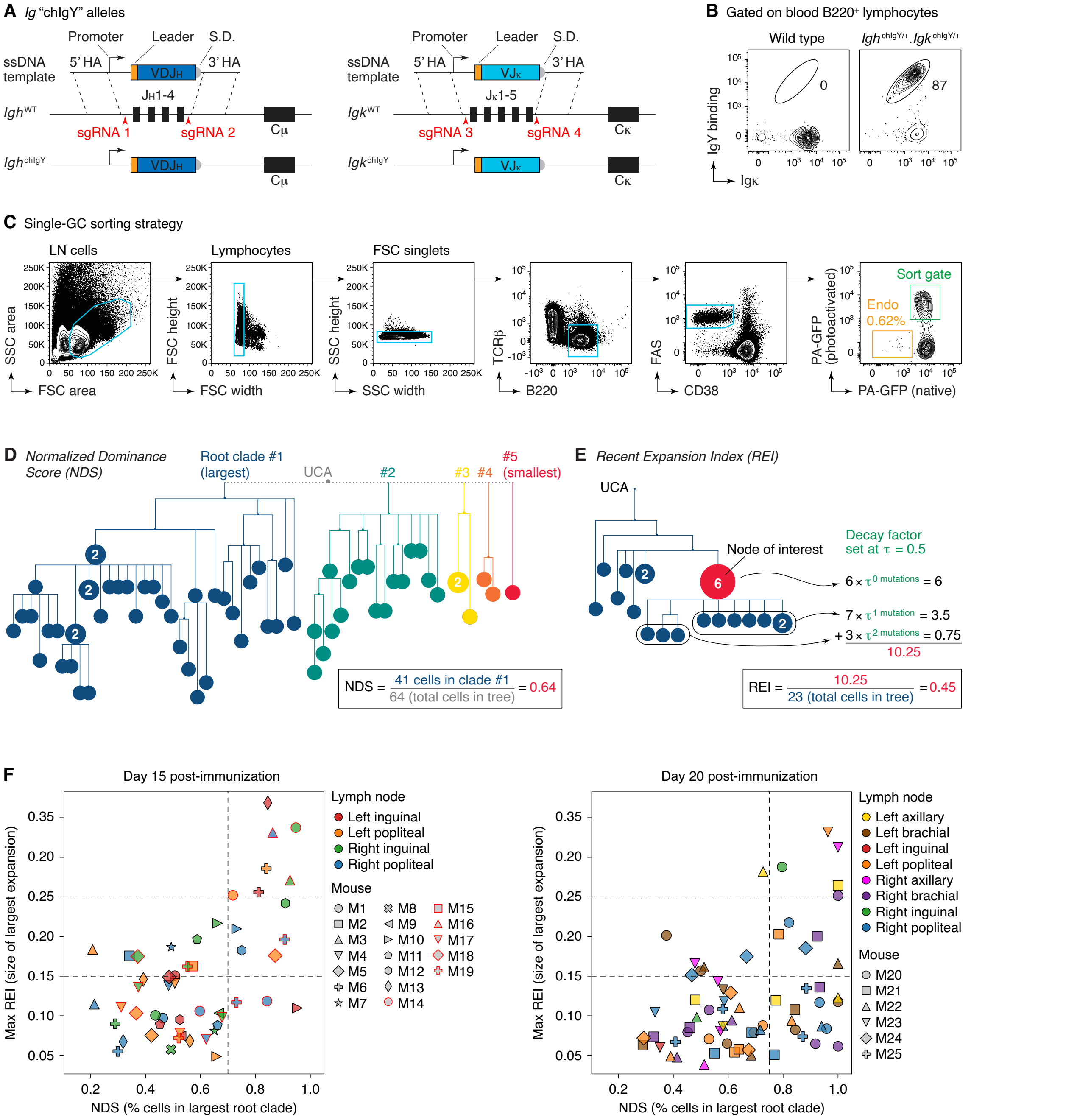

Figure S1. Parallel replay of germinal center evolution (related to Figure 1).

(A) Design of the *Igh* and *Igk* "chIgY" alleles. CRISPR/Cas9 genome targeting with single-stranded DNA templates was used to replace the endogenous J_H and J_κ segments with pre-rearranged V(D)J genes. HA, homology arm; S.D., splice donor.

(B) Flow cytometry of blood B cells from wild-type and chIgY mice, showing expression of the rearranged V(D)J genes (inferred from the ability of B cells to bind IgY).

(C) Sorting strategy for the parallel replay experiment. FACS plots show a representative LN fragment. Endo, residual endogenous GC B cells derived from the CD23-Cre.*Bcl6*$^{flox/flox}$ host.

(D) Schematic representation of the normalized dominance score (NDS) calculation. NDS is equal to the percentage of all cells in a GC that belong to the largest root clade (dark blue). The size of the smaller clades is not included in the calculation. This metric is loosely equivalent to the color-based NDS used to quantify clonality in “Brainbow” experiments[7].

(E) Schematic representation of the recent expansion index (REI) calculation. For each node X in a phylogeny, the REI represents the sum of the number of descendants of node X weighted according to their mutational distance from node X using a decay factor τ = 0.5, such that cells at 0, 1, 2, ... nucleotide distance from node X are weighted 1, 0.5, 0.25, … (this decay factor is based on the observation that clonal bursting involves a transient cessation of SHM[42,96]); the sum of weighted descendants is then divided by the total number of cells in the GC. A phylogeny in which all cells have the same sequence therefore has an REI of 1.0. For each GC, we plot the value for its highest-REI node (max REI).

(F) Distribution of NDS and REI scores for GCs from 15 and 20 dpi as in Fig. 1E,F, but colored by mouse and LN of origin; each symbol represents one GC.

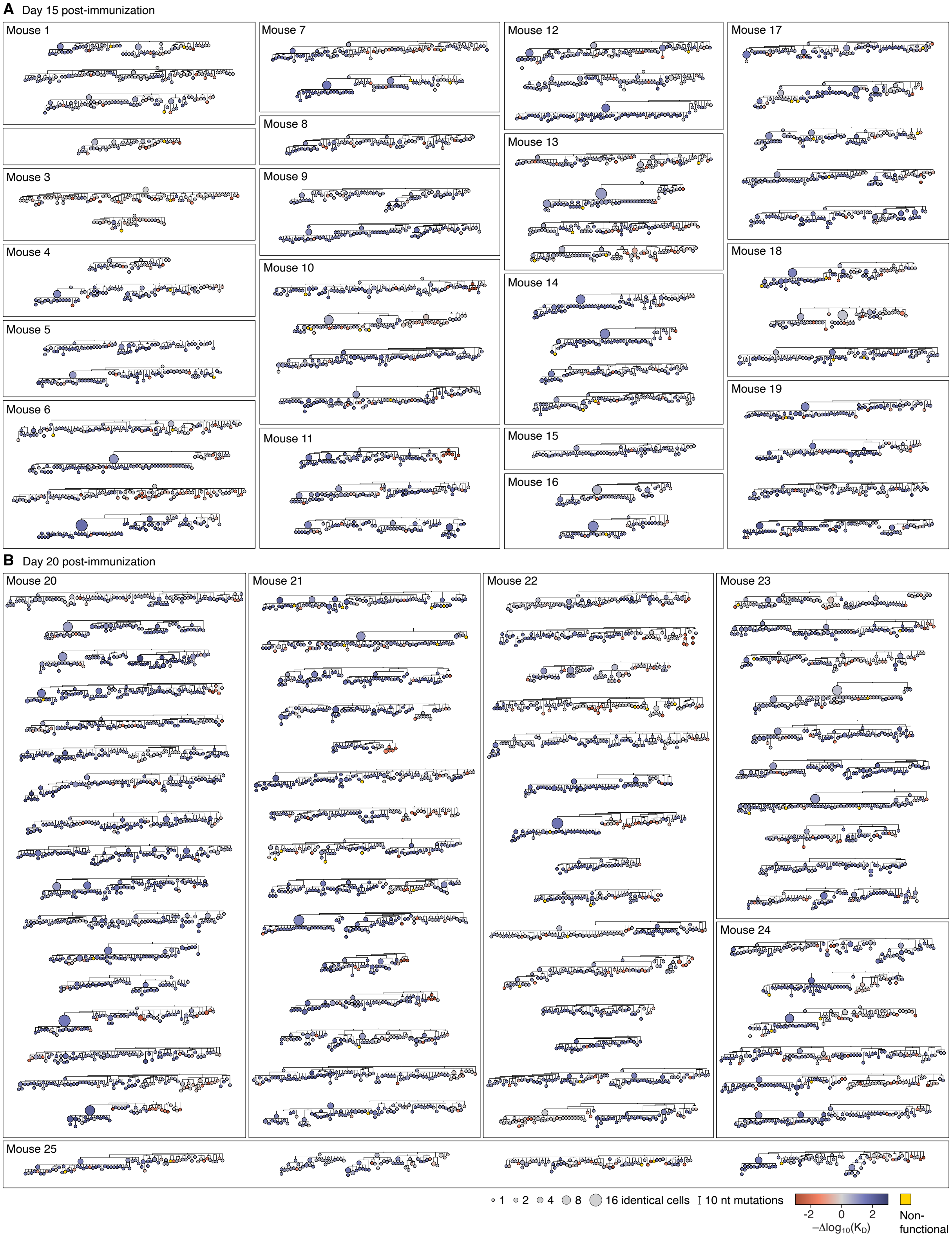

Figure S2. Individual germinal center phylogenies (related to Figure 1).

Diagrams of all 119 phylogenetic trees inferred from the *Igh+Igk* sequences obtained from each photoactivated GC at (A) 15 and (B) 20 days post-immunization. Boxes indicate the mouse from which each GC was sorted. Nodes are colored by their relative affinity from the naive precursor (–Δlog10(K_D)), see Fig. 2 for details.

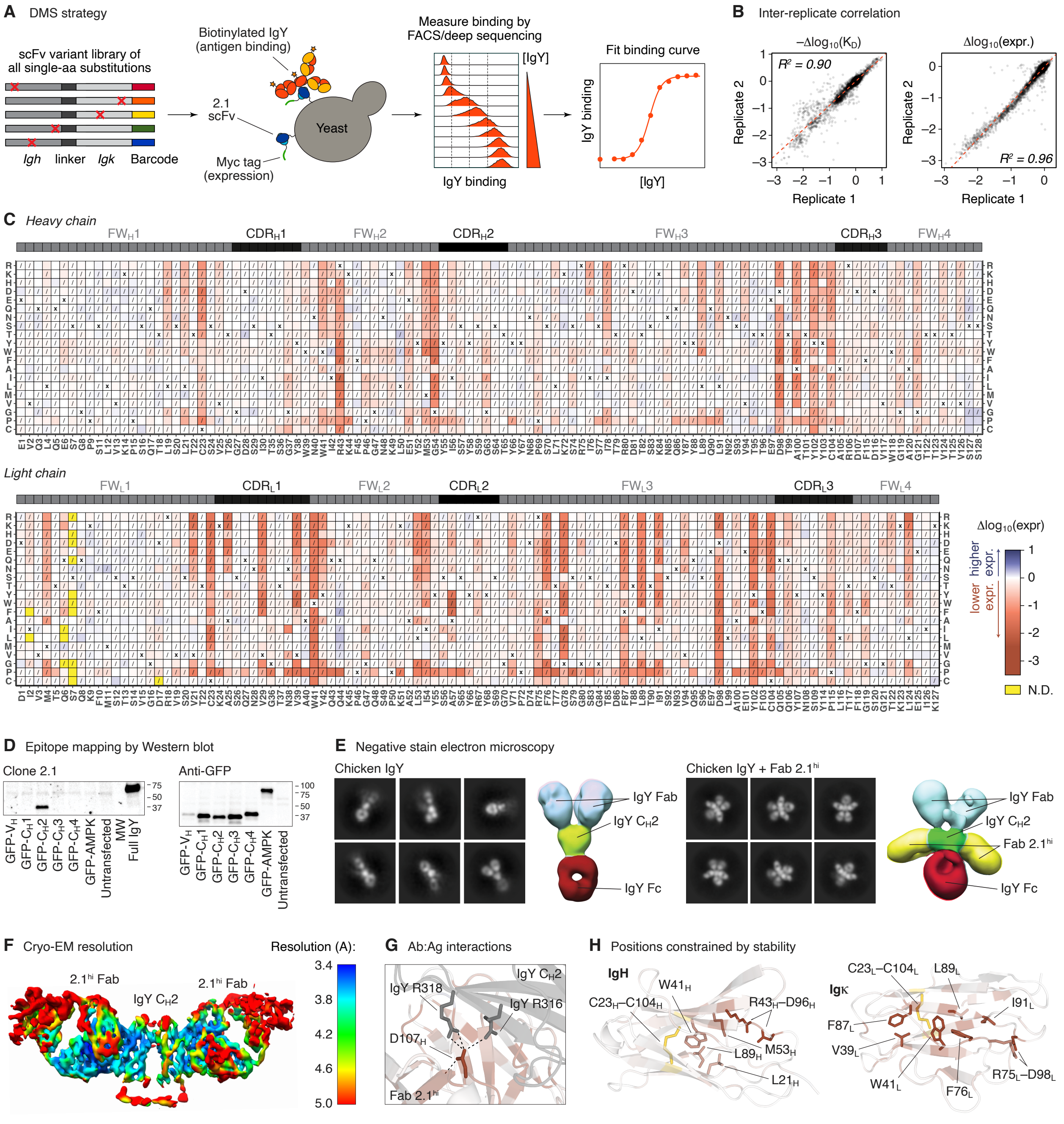

Figure S3. Deep mutational scanning and structure of clone 2.1 (related to Figure 2).

(A) Experimental setup for the DMS experiment.

(B) Correlation in mutation effects on CGG binding affinity (left) and scFv expression level (right) from independently generated and assayed mutant libraries.

(C) Heatmap showing the effects of individual amino-acid replacements on surface expression of 2.1 scFv by yeast display. Each square represents a different replacement. Squares marked with “X” indicate the original aa in clone 2.1. Squares with slashes indicate aa replacements >1 nucleotide mutation away from the naïve sequence. Yellow squares were not detected in the DMS experiment.

Upper bar shows Kabat CDR and framework (FW) regions in black and gray, respectively. An interactive version of this heatmap is available at https://matsengrp.github.io/gcreplay/interactive-figures/mutation-heatmaps/naive_reversions_first.html.

(D) Coarse mapping of the epitope of clone 2.1. 293T cells were transfected with constructs encoding each Ig domain of IgY_H (or AMPK as a control) fused to GFP. Cell extracts were probed by Western blot with recombinant clone 2.1 mAb (left) or with polyclonal anti-GFP to detect expression of the construct (right). MW, molecular weight ladder, not visible by Western blot.

(E) Negative-stain 2D classes and 3D reconstructions (colored by domain/subunit) of unliganded chicken IgY (left) and the 2.1^{hi} Fab:IgY complex (right) confirming Fab binding to C_H2.

(F) Cryo-EM reconstruction of the 2.1^{hi} Fab:IgY complex colored by local resolution following IgY C_H2 and 2.1^{hi} Fab local refinement.

(G) Key inferred electrostatic interaction between clone 2.1 and IgY. Backbone and side chains colored by mean ΔK_D for all replacements at each position. Color scale as in Fig. 2A. Potential salt bridge interactions are shown as dotted lines. Mutation of $D107_H$ to anything other than an acidic aa ($D107_HE$) results in at least 1 $\log_{10}$ decrease in binding affinity.

(H) Mapping of selected residues that strongly affect antibody surface expression when mutated. Backbone and side chains colored by mean Δexpression for all replacements at each position. Color scale as in (C); disulfide bridges shown in yellow.

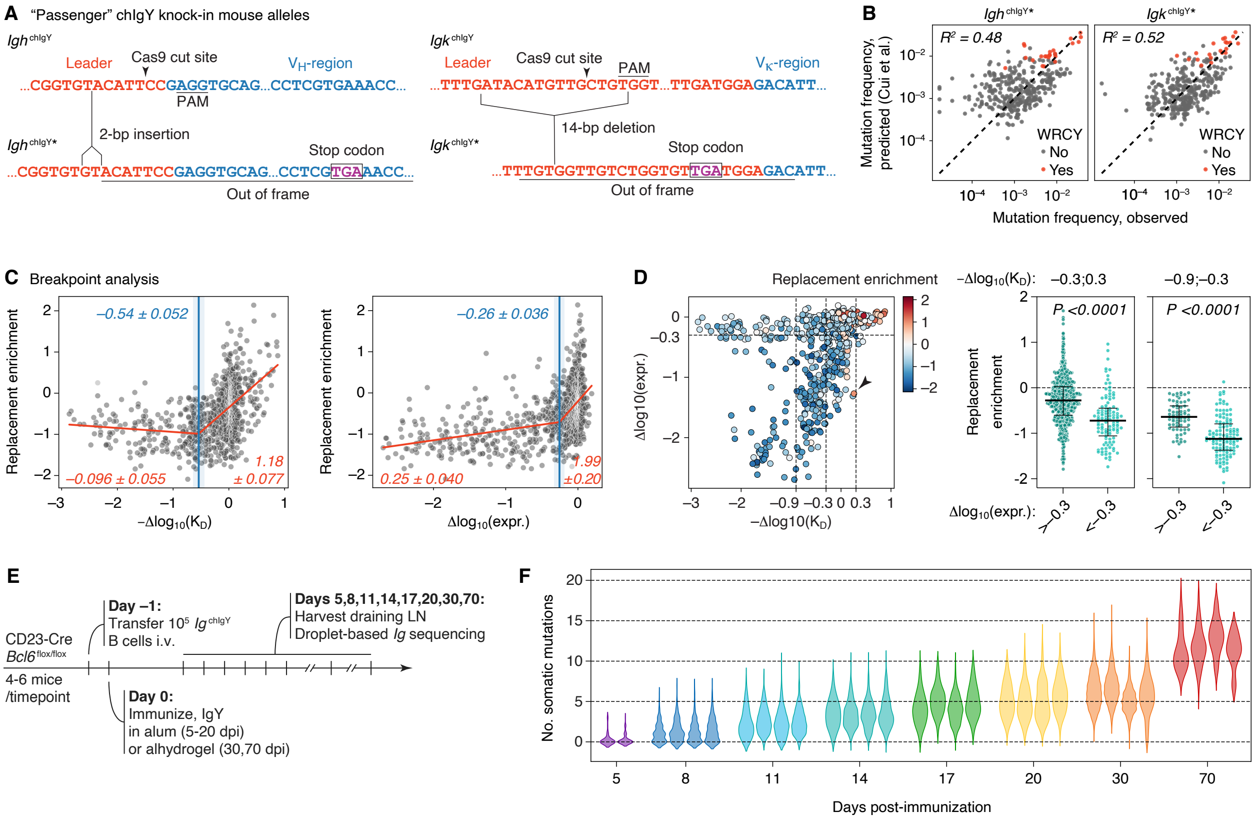

Figure S4. Selection of individual aa replacements (related to Figure 3).

(A) Sequences of the "passenger" *Igh*^{chIgY*} and *Igk*^{chIgY*} alleles generated by CRISPR/Cas9-mediated cleavage on the *Ig*^{chIgY} background.

(B) Comparison of relative mutation frequencies observed in passenger allele mice *in vivo* with predictions made using the five-mer model[23]. Each symbol represents one nucleotide position of the respective *Ig* sequence. C/G pairs within RGYW AID hotspot motifs are highlighted in red.

(C) Segmented regression analysis using the piecewise-regression package in Python. A two-segment fit was chosen by model comparison, and the results of that fit are shown here.

(D) Each replacement, plotted in terms of its effect on affinity and expression, and colored according to replacement enrichment. Arrowhead indicates an exceptional replacement that is enriched even though it leads to a marked loss of expression.

(E) Layout of the time-course experiment.

(F) Distribution of somatic mutations at the indicated time points post-immunization. Each violin represents one mouse. Data for 5-20 dpi were obtained together in a single experiment; data for 70 dpi is from a separate experiment. Two mice (of a total of 9) were excluded from the 70 dpi violin plots for insufficient cell yield; cells from these mice were included in the bulk analysis.

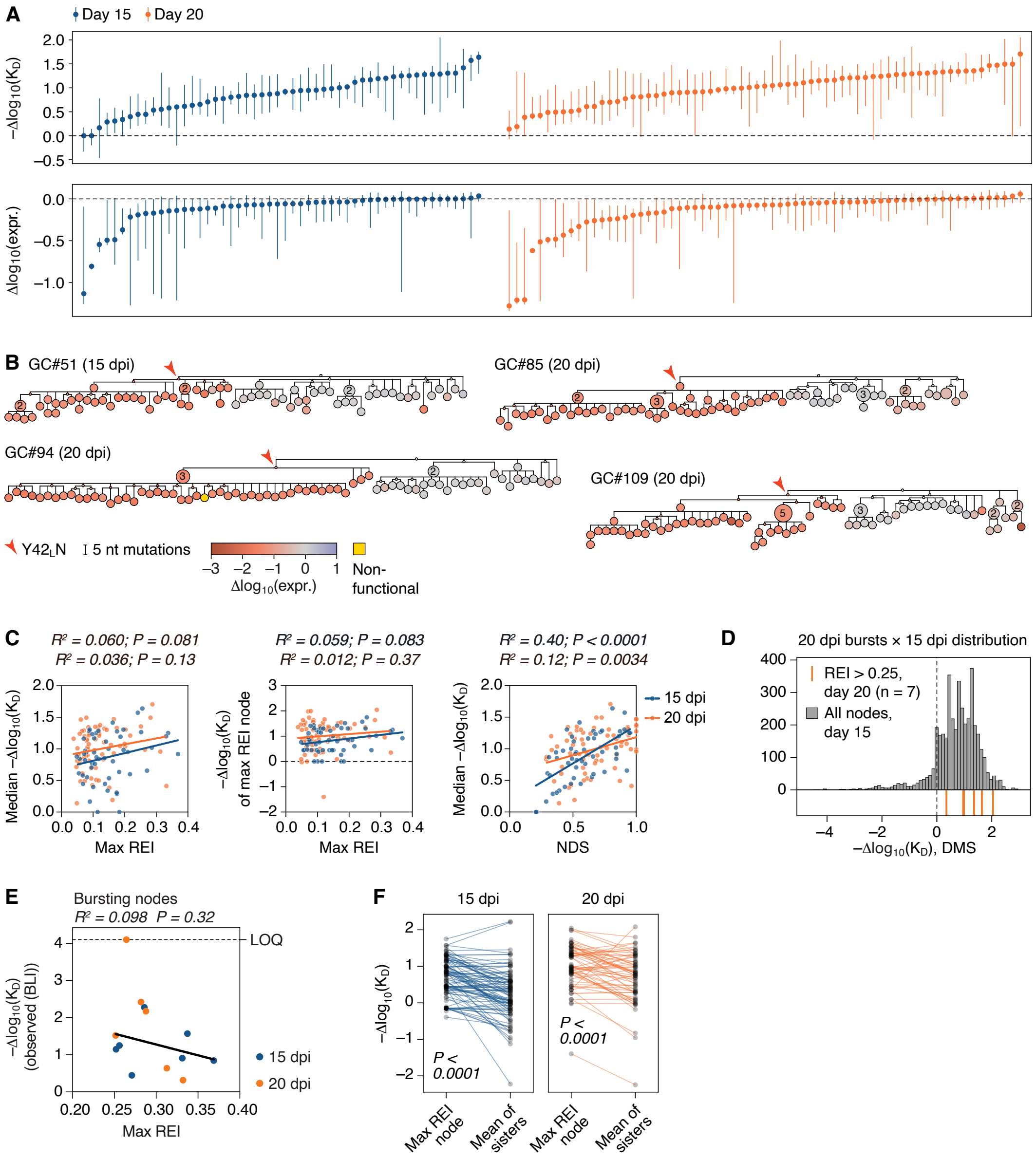

Figure S5. Drivers of affinity maturation and maintenance of Ig expression (related to Figures 4 and 5).

(A) Median (± range) of Δaffinity (top) and Δexpression (bottom) for all GCs in the replay experiment. Each symbol represents one GC.

(B) Examples of GC phylogenies carrying the expression-impairing $Y42_LN$ replacement. Trees are colored by Δexpression.

(C) Correlations between phylogenetic selection parameters for each GC (max REI and NDS) and median Δaffinity or the Δaffinity corresponding to the sequence of the max REI node). Each symbol represents one GC. Trend lines are for each time point. R^2 and P-values are for Pearson correlation and are given for 15 dpi (top row) and 20 dpi (bottom row).

(D) Overlay of the distribution of Δaffinities for clonal burst nodes obtained at 20 dpi (REI >0.25; orange lines) compared to the distribution of Δaffinities for all nodes (observed and inferred) from 15 dpi (grey bars).

(E) Correlation between BLI-measured Δaffinities and the Max REI in the GC (corresponding to the REI of the sequence of the bursting B cell itself) of each of the bursting nodes in Fig. 5D. Solid black line is the linear trend. LOQ, limit of quantitation, when Fab off-rate is too long to be determined. R^2 and slope p-value calculations exclude the Fab with Δaffinity >LOQ.

(F) Statistical comparison of the affinity of the max REI node in each replay GC and the mean affinity of its “sister” nodes, as defined in Fig. 5G. P-values are for the Wilcoxon signed-rank test.

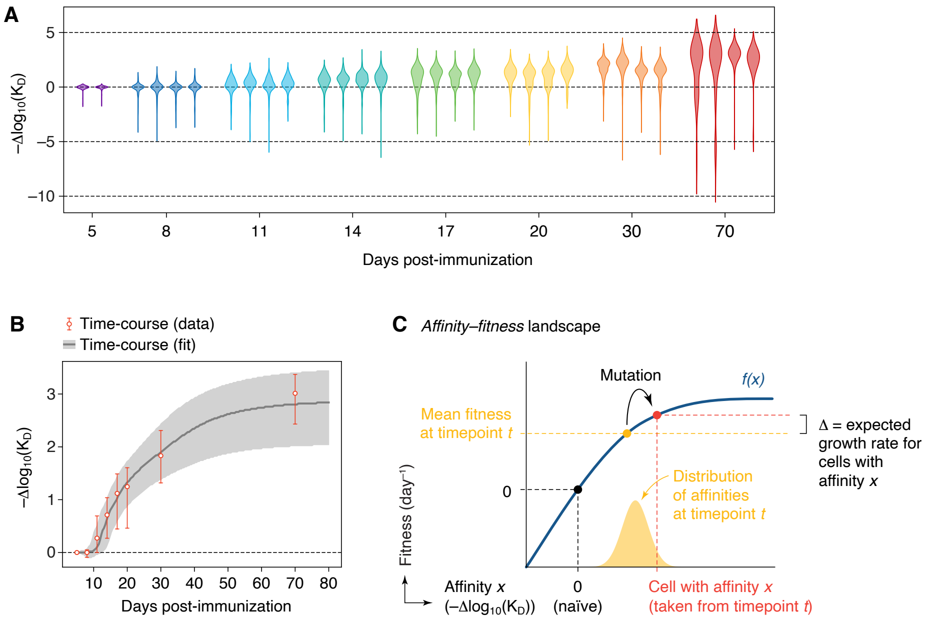

Figure S6. Progression of affinity over time in bulk-sorted GC B cells (related to Figure 6).

(A) Distribution of DMS-inferred affinities at the indicated time points post-immunization. Each violin represents one mouse. Data for 5-20 dpi were obtained together in a single experiment; data for 70 dpi is from a separate experiment. Two mice (of a total of 9) were excluded from the 70 dpi violin plots for insufficient cell yield and a further three were excluded due to prominent subpopulations of low affinity cells (which suggest co-option of GC lineages to bind other antigens[103,104] in a host mouse strain unable to generate GCs of its own).

(B) Given the consistency of affinity distributions across individual mice, we aggregated cells from each time point to approximate a single longitudinal sequence of affinity distributions sampled throughout the time-course. Graph shows median and IQR of this distribution overlaid on the prediction generated by the fitness landscape model.

(C) Schematic representation of the affinity–fitness landscape, adapted from Held et al.[44] This landscape translates changes in affinity (X-axis) into changes in “evolutionary fitness” (Y-axis), corresponding roughly to the expansion rate expected for a population of B cells with affinity X when compared to a naïve competitor population with affinity = 0, in the absence of further mutation. Operationally, the difference (Δ) between the fitness of a population of B cells with affinity *x* sampled at time-point *t* (red) and the mean fitness of all cells at time point (orange) corresponds to the expected exponential growth rate (or decay rate, when negative) for that population over time. The detailed shape of the function *f(x)* specifies how fitness responds to marginal affinity increases and how this responsiveness attenuates as one moves up in the affinity axis. Mathematical details of the model are elaborated in the Methods.

Supplemental Table 1. Data collection, processing, model refinement and validation

**Map**	**IgY + Clone 2.1 Fab (local refinement)**
EMDB	EMD-70353
**Data collection**	
Microscope	Thermo Fisher Talos Arctica
Voltage (kV)	200
Detector	Gatan K2 Summit
Recording mode	Counting
Nominal magnification	36,000x
Movie micrograph pixelsize (Å)	1.15
Dose rate (e^-/[(camera pixel)*s])	6.95
Number of frames per movie micrograph	47
Frame exposure time (ms)	200
Movie micrograph exposure time (s)	9.5
Total dose (e^-/Å^2)	50
Defocus range (μm)	-0.8 to -2.5
**EM data processing**	
Number of movie micrographs	10,007
Number of molecular projection images in map	346,259
Symmetry	C1
Map resolution (FSC 0.143; Å)	4.0
Map sharpening B-factor (Å^2)	-171
**Structure building and validation**	
Number of atoms in deposited model	
IgY CH2	1,434
Clone 2.1 Fab	3,400
glycans	28
MolProbity score	1.22
Clashscore	1.36
Map correlation coefficient	0.73
EMRinger score	1.63
d FSC model (0.5; Å)	4.3
RMSD from ideal	
Bond length (Å)	0.007
Bond angles (˚)	1.203
Ramachandran plot	
Favored (%)	94.96
Allowed (%)	5.04
Outliers (%)	0.00
Side chain rotamer outliers (%)	0.00
Cβ outliers (%)	0.00
PDB	9ODB
